# Open questions on the mechanisms of neuromodulation with applied and endogenous electric fields

**DOI:** 10.3389/fnhum.2014.00227

**Published:** 2014-04-17

**Authors:** Shennan A. Weiss, Marom Bikson

**Affiliations:** ^1^Neurology, Columbia UniversityNew York, NY, USA; ^2^Biomedical Engineering, The City College of New York of CUNYNew York, NY, USA

**Keywords:** ephaptic, transcranial direct current stimulation (tDCS), transcranial magnetic stimulation, brain oscillation, stimulation, systems neuroscience

## Introduction

Despite a long-standing recognition that bioelectric phenomena underpin brain function, fundamental questions remain about how extracellular current flow may influence neural activity and computation. The source of extracellular current flow may be exogenous, electrical exposure or stimulation, or the source may be endogenous, for currents produced by the brain itself. The former has recently gained increased urgency with the evolution of transcranial electrical therapy for a broad range of neurological and psychiatric disorders. The latter remains one of the longest standing open questions in neuroscience—is the electrical current flow that is a ubiquitous aspect of brain function (manifest for example in oscillations, EEG) an epiphenomenon or a key functional signal in the brain.

Field effects that are produced by transmembrane currents are called ephaptic. An ephapse “*Gr*: touching across” was originally coined to describe how two axons placed close together in mineral oil, which has a higher resistance than saline, transmit an action potential at what can be considered an artificial synapse (Katz and Schmitt, 1940; Arvanitaki, 1942). This finding led Sir John Eccles to propose the Golgi cell theory of inhibition in which he speculated that ephapses could mediate inhibitory neurotransmission (Brooks and Eccles, 1947). Eccles corrected this theory and was awarded a Nobel Prize for his subsequent work demonstrating that inhibitory neurotransmission is mediated by chemical synapses. However, in accord with Eccles original theory, ephaptic transmission has been found to mediate inhibitory neurotransmission at the Mauthner cell axon hillock (Weiss et al., 2008), and the Pinceau of the cerebellar Purkinje cell (Korn and Axelrad, 1980; Blot and Barbour, 2014). Research during the twenty-first century has demonstrated that field effects in the mammalian brain may be much more ubiquitous. Field effects generated by endogenous activity may influence network oscillations and computation throughout the cerebral cortex (Radman et al., 2007). Both physiologic (e.g., oscillations; Parra and Bikson, 2004) and pathologic (e.g., epilepsy; Haas and Jefferys, 1984) activity may be influenced by field effects. Because field effects are both generated by coherent population activity and influence networks in a coherent fashion, they may influence brain function as no neurotransmitter can.

Furthermore, weak direct and alternating current stimulation of the human cerebral cortex at low-intensity strengths have been found to influence network dynamics and behavior. The exploration of transcranial Direct Current Stimulation (tDCS) and transcranial Alternative Current Stimulation (tACS) over the past decade for both treatment and to enhance cognitive performance and learning in healthy individuals, has galvanized questions about how the brain responds to low-intensity stimulation. Indeed, the intensity of electric generated in these modalities can approximate the intensity of electricity generated by the bran itself (Datta et al., 2009).

Thus the science of field-effects and low-intensity electrotherapy overlap. The articles included in this e-book highlight some of the latest developments in understanding both endogenous field effects in the central nervous system, as well as the mechanisms and clinical applications of transcranial stimulation of the cortex. We hope that these articles are helpful for students, researchers, and clinicians who hope to better understand and utilize this often overlooked form of neurotransmission.

### Conflict of interest statement

The authors declare that the research was conducted in the absence of any commercial or financial relationships that could be construed as a potential conflict of interest.
